# A phase III, multi-centre, double-masked randomised controlled trial of adjunctive intraocular and peri-ocular steroid (triamcinolone acetonide) versus standard treatment in eyes undergoing vitreoretinal surgery for open globe trauma (ASCOT): statistical analysis plan

**DOI:** 10.1186/s13063-016-1464-4

**Published:** 2016-08-02

**Authors:** Jessica W. Lo, Catey Bunce, David Charteris, Philip Banerjee, Rachel Phillips, Victoria R. Cornelius

**Affiliations:** 1 Department of Primary Care and Public Health Sciences, King’s College London, 4th Floor Addison House, Guy’s Campus, London, SE1 1UL UK; 2 Moorfields Eye Hospital NHS Foundation Trust, City Road, London, EC1V 2PD UK; 3 National Institute for Health Research (NIHR) Biomedical Research Centre for Ophthalmology at Moorfields Eye Hospital NHS Foundation Trust and UCL Institute of Ophthalmology, 162 City Road, London, EC1V 2PD UK; 4 National Institute for Health Research (NIHR) Clinical Research Facility at Moorfields Eye Hospital, London, UK; 5 National Institute for Health Research (NIHR) Biomedical Research Centre at Guy’s and St. Thomas’ NHS Foundation Trust and King’s College London, London, UK; 6 King’s Clinical Trials Unit, Institute of Psychiatry, King’s College London, London, UK; 7 Imperial Clinical Trials Unit, School of Public Health, Imperial College London, Stadium House, 68 Wood Lane, London, W12 7RH UK

**Keywords:** Statistical analysis plan, Randomised controlled trial, Retina, Open globe trauma, Triamcinolone acetonide

## Abstract

**Background:**

Open globe ocular trauma complicated by intraocular scarring (proliferative vitreoretinopathy) is a relatively rare, blinding, but potentially treatable condition for which, at present, surgery is often unsatisfactory and visual results frequently poor. To date, no pharmacological adjuncts to surgery have been proven to be effective. The aim of the Adjunctive Steroid Combination in Ocular Trauma (ASCOT) randomised controlled trial is to determine whether adjunctive steroid (triamcinolone acetonide), given at the time of surgery, can improve the outcome of vitreoretinal surgery in patients with open globe ocular trauma. This article presents the statistical analysis plan for the main publication as approved and signed off by the Trial Steering Committee prior to the first data extraction for the Data Monitoring Committee meeting report.

**Methods/design:**

ASCOT is a pragmatic, multi-centre, parallel-group, double-masked randomised controlled trial. The aim of the study is to recruit from 20–25 centres in the United Kingdom and randomise 300 eyes (from 300 patients) into two treatment arms. Both groups will receive standard surgical treatment and care; the intervention arm will additionally receive a pre-operative steroid combination (triamcinolone acetonide) into the vitreous cavity consisting of 4 mg/0.1 ml and 40 mg/1 ml sub-Tenon’s. Participants will be followed for 6 months post-surgery. The primary outcome is the proportion of patients achieving a clinically meaning improvement in visual acuity in the study eye at 6 months after initial surgery, defined as a 10 letter score improvement in the ETDRS (the standard scale to test visual acuity).

**Trial registration:**

ISRCTN30012492. Registered on 5 September 2014.

EudraCT2014-002193-37. Registered on 5 September 2014.

## Background

Trauma is a major cause of visual impairment and blindness worldwide; in particular, it is the most common cause of unilateral blindness [1, 2]. Approximately 1.6 million people across the globe became blind as a result of ocular trauma, with up to 19 million living with unilateral blindness or low vision [2]. Ocular injuries invariably affect the posterior segment of the eye, and vitreoretinal surgery is required to prevent visual loss. Recent published results have shown that, although vitreoretinal surgical techniques have improved, outcomes remain unsatisfactory, mainly due to the development of the intraocular scarring response proliferative vitreoretinopathy (PVR) [3–6]. PVR is the main cause of recurrent retinal detachment and visual loss in eyes with open globe trauma (OGT). Although final retinal attachment may now be achieved, multiple surgeries are often needed, and visual results remain very poor [7, 8]. PVR is a difficult vitreoretinal surgical challenge, and its management is costly in both patient time and healthcare resources [8]. To date, no pharmacological adjuncts to surgery have been proven to be effective in treating OGT complicated by PVR. Experimental work has suggested that steroid (triamcinolone acetonide) treatment can reduce the severity of PVR [9] and that it appears to have no significant retinal toxicity [10].

### Objective

The primary objective of the Adjunctive Steroid Combination in Ocular Trauma (ASCOT) trial is to determine whether adjunctive triamcinolone acetonide, given at the time of surgery, can improve visual acuity (VA) at 6 months compared with treatment as usual in eyes of patients undergoing vitreoretinal surgery for OGT. Additionally, the influence of the intervention on the development of scarring (PVR), as well as the incidence of retinal detachment, intraocular pressure (IOP) abnormalities and other complications in eyes undergoing surgery for OGT, will be examined. Quality of life measured using the 25-item Visual Function Questionnaire (VFQ-25) will also be assessed.

## Methods/design

ASCOT is a pragmatic, multi-centre, double-masked randomised controlled trial. Three hundred adult participants with OGT will be recruited from 20–25 centres across the United Kingdom. The inclusion and exclusion criteria are listed below.

### Inclusion criteria

1. Adult subjects aged 18 years or older at the time of enrolment
2. Full-thickness OGT undergoing vitrectomy
3. Ability to give written informed consent
4. Willingness to accept randomisation and attend follow-up for 6 months

### Exclusion criteria

1. Pre-existing uncontrolled uveitis
2. Definitive diagnosis of previous steroid-induced glaucoma
3. Pregnant or breastfeeding females
4. Allergy or previous known adverse reaction to triamcinolone acetonide
5. Current or planned systemic corticosteroid use at a dose above physiological levels (e.g., >10 mg prednisolone)

Participants will be individually randomised in a 1:1 ratio to surgery plus adjunctive triamcinolone acetonide or surgery only. Randomisation will be carried out by permuted blocks with stratification by trial centre conducted using a telephone service to the Emergency Scientific Medical Services global service hosted at the King’s Clinical Trials Unit (KCTU) at King’s College London. Randomisation and subsequent treatment allocation will be performed intraoperatively once the operating surgeon has confirmed that the retina is attached. The participants in the intervention arm will receive pre-operative steroid combination (triamcinolone acetonide) into the vitreous cavity of the study eye, consisting of 4 mg/0.1 ml and 40 mg/1 ml sub-Tenon’s; the standard treatment group will receive standard care only (surgery without adjunctive treatment). The study eye is defined as the eye which requires vitrectomy. It is extremely rare for patients to experience bilateral OGT requiring surgery in both eyes; however, for these cases, the surgeon will select the worse eye (i.e., the eye with the least good potential visual outcome, based on the pre-operative assessment of ocular and, in particular, macular trauma) to be the study eye. Patients will be followed and assessed at 3 months and 6 months post-surgery, in line with the usual clinical follow-up appointments. Operating surgeons will be masked until the end of surgery (when the adjunct is given); patients and study investigators will be masked throughout, except for one of the trial statisticians, who will be masked to sub-group and will undertake analyses for reports to the data monitoring committees. Ethics approval was granted by the Central London NHS Research Ethics Committee. All patients are to provide written informed consent prior to randomisation.

### Outcomes

#### Primary outcome

The primary outcome is defined as the proportion of patients with a clinically meaningful improvement in VA in the study eye (yes or no). A meaningful improvement in VA is defined as having an improvement in VA of 10 letters or more in the study eye and is calculated as the difference between ETDRS score (Early Treatment Diabetic Retinopathy Study, the standard scale to test VA, which is based on letters of decreasing size on a chart) measured at baseline and 6-month follow-up [11]. The ETDRS score is measured by using the ETDRS vision chart at a starting distance of 4 m. The patient starts from the top of the chart and reads down the chart, and the score is the sum of the number of letters that could be correctly identified. The ETDRS score will be dichotomised into two groups: patients with a change in VA <10 and patients with a meaningful improvement in VA (change in VA ≥10 letters).

The primary outcome was chosen to be analysed as a binary variable instead of a continuous one for the following reasons: (1) the binary outcome represents a clinically more meaningful and tangible result for patients and clinicians that can be easily communicated, and (2) the results of the ASCOT pilot study demonstrated a non-identical distributional shift in ETDRS letter score at follow-up between the two treatment arms. This resulted in a small mean difference in ETDRS scores between treatment arms potentially masking a clinically important difference in the proportion of participants with a meaningful improvement in VA between treatment arms.

#### Secondary outcomes

The secondary outcomes are described below:

1. ETDRS letter score as measured by ETDRS vision charts at 6-month follow-up (analysed as a continuous outcome)
2. The proportion of patients in whom retinal detachment with PVR occurs at any time point within 6 months of the study vitrectomy
3. The proportion of patients in whom stable, complete retinal reattachment (without internal tamponade present) is achieved at 6 months after study vitrectomy
4. The proportion of patients in whom stable macular retinal reattachment (without internal tamponade present) is achieved at 6 months after study vitrectomy
5. The proportion of patients in whom a tractional retinal detachment occurs at any time point within 6 months of the study vitrectomy
6. The number of operations needed to achieve stable retinal reattachment (either complete or macular) at 6 months after the study vitrectomy
7. The proportion of patients with hypotony (<6 mmHg) at any time point within 6 months of the study vitrectomy
8. The proportion of patients with raised IOP (>25 mmHg) at any time point within 6 months of the study vitrectomy
9. The proportion of patients who develop macular pucker by 3 and 6 months and/or require macular pucker surgery at any time point within 6 months of the study vitrectomy
10. Quality of life at 6 months after the study vitrectomy based on the following:
  - Client Service Receipt Inventory (CSRI)
  - EQ-5D-5L questionnaire
  - VFQ-25

Detailed analysis plans for these health economic assessments are documented separately by the trial’s health economist. Note that all eye-related outcomes relate to the study eye.

#### Sample size calculation

With 90 % power and 5 % significance (two-sided test), a sample size of 140 per group will be required to detect a 19 % increase (from 55 % to 74 %) in the proportion of participants with a minimum improvement in VA of ≥10 EDTRS letter score. The target sample size has been inflated to 300 to allow for a 7 % dropout rate.

### Statistical analyses

#### Trial profile

**Fig. 1:**
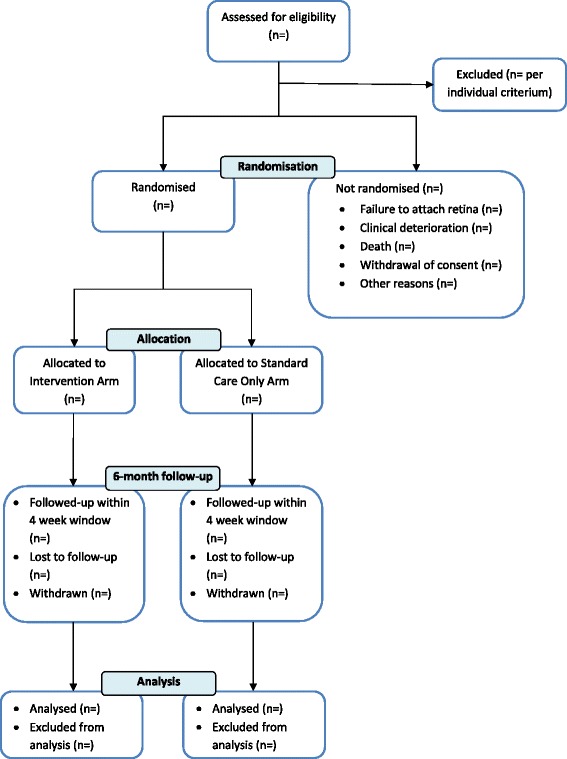
Consolidated Standards of Reporting Trials (CONSORT) trial flow diagram

The flow of participants will be displayed in a Consolidated Standards of Reporting Trials (CONSORT) diagram, as shown in Fig. 1. The number of protocol deviations with reasons will be reported.

### Data management and quality assurance

Study data will be initially recorded on worksheets and then entered, by designated research personnel in each study centre, into an online database (InferMed MACRO; Elsevier, London, UK) hosted at the KCTU at King’s College London. Research coordinators at each site and the trial manager will periodically perform basic checks, such as examining for improbable values and data completeness. Errors will be explored and checked with original source data. After the last patient is followed, all queries resolved and all data fields completed with data or missing data codes, the database will be locked for final analysis, and this process will be overseen by KCTU.

#### General analysis principles

The analytical principles outlined will be followed as closely as possible in the analysis and reporting of trial data. The statistical analysis plan is not intended to restrict exploratory or other sensible and standard reporting practices. There are no plans for any formal comparisons between treatment arms until final database lock. Analysis will be undertaken by the trial statistician, who is masked to sub-groups. Group allocation will be revealed to the study team after the study analytical report has been constructed.

Missing outcome data are anticipated to be low, and the sample size includes provision for withdrawal and loss to follow-up. The main analyses for the primary and secondary outcomes will follow the intention-to-treat principle: All randomised participants with primary outcome data will be analysed in their assigned treatment groups, regardless of the treatment they actually received. A sensitivity analysis examining assumptions regarding missing outcome data will be undertaken. (See below for a discussion on missing data.) As the intervention occurs immediately after randomisation, the safety population will consist of all randomised participants unless it is documented that they did not receive the intervention. All statistical tests and confidence intervals will be two-sided, and the significance level is set at 5 %.

#### Baseline characteristics

All baseline characteristics (see Appendix 1) will be summarised by randomised group. Summary measures for the baseline characteristics of each group will be presented as mean and SD for continuous, approximately normally distributed variables; medians and interquartile ranges will be used for non-normally distributed variables; and frequencies and percentages will be used for categorical variables. These summaries will be based on observations only, and the number of missing observations for each characteristic will be reported.

For all outcomes, a generalised linear model (GLM) that includes treatment arm, baseline value and centre as covariates will be fitted. As the primary outcome is binary, if there are many centres enrolled that recruit only a small number of participants, a sensitivity analysis exploring suitable methods to adjust for centre in the model will be undertaken [12].

#### Analysis of primary outcome

The proportion of participants with improvement in VA ≥10 letters (yes or no) will be tabulated by treatment arm and time point. Initially, an unadjusted difference in proportion at 6 months between treatment arms will be calculated with 95 % confidence intervals. The adjusted treatment effect estimate will be obtained by fitting a GLM with improvement in VA ≥10 letters (yes or no) as the outcome, and study arm and baseline VA scores as the covariates. The treatment effect estimate will be reported with a two-sided 95 % confidence interval and corresponding *p* value. A GLM with binomial distribution and logit link function will be used, and the odds ratio will be reported with 95 % confidence interval.

#### Sensitivity analyses for primary outcome

As the intervention is a one-off treatment at the time of randomisation and the two follow-up appointments follow usual clinical care, we anticipate a low percentage of missing primary outcome data. It is anticipated that missing data will be missing at random (MAR). A sensitivity analysis of the primary outcome will be undertaken to assess the impact of participants with missing VA scores at 6-month follow-up. The number, pattern and timing of missing data will be examined by treatment arm along with the reasons for withdrawal or for missing data. Potential bias due to missing data will be investigated initially by comparing the baseline characteristics (using descriptive comparisons) between participants with complete follow-up measurements and those without primary outcome data. A missing indicator variable (yes or no) will be generated for data at 6 months, and the relationship between study variables and missingness will be examined using logistic regression. On the basis of our exploration of missing data, we will consider whether our assumption of MAR is reasonable.

Due to the anticipated small number of missing data and the binary primary outcome, we will initially undertake an extreme value analysis to examine the impact of missing data. The sensitivity analysis will consider optimistic and pessimistic scenarios for patients in both treatment arms. Our missing data analysis will be complete if we find the results are consistent with the primary analysis. If the results include a range of values inconsistent with the primary analysis we will undertake a sensitivity analysis with more plausible assumptions using multiple imputation. Datasets will be imputed using the method described by Carpenter and Kenward [13]. If there is an unexpectedly large number of participants lost to follow-up and we consider that the data may be missing not at random, then we will examine the impact of this using multiple imputation with a weighting approach described by Carpenter, Kenward and White [14].

#### Sub-group analysis

Sub-group analysis will be performed for the primary outcome to explore the uniformity of the treatment effects found overall. The following sub-groups will be examined:

1. *Retinal detachment*: Attached, traction retinal detachment and rhegmatogenous retinal detachment
2. *Foveal involvement*: Yes, no and splitting
3. *Presence of PVR*: Yes and no
4. *Presence of retinal incarceration*: Yes and no
5. *Lens status at baseline*: Clear (phakic), cataract (phakic), pseudophakic (anterior chamber and posterior chamber intraocular lens) and aphakic

Each sub-group analysis will be performed by adding the relevant treatment-by-sub-group interaction term to the same analytical model used for the primary outcome. *p* Values for each interaction term will be presented. No adjustment for multiple tests will be made, and the results will be viewed as hypothesis-generating only. The consistency of estimates will be depicted visually by means of a forest plot.

#### Analysis of secondary outcomes

Analysis of secondary outcomes will be undertaken using a similar approach to that described for the primary analysis. Secondary outcomes will be summarised and tabulated by treatment arm and time point. We will estimate and test for a difference between treatment arms for each endpoint specified in the secondary outcomes listed above. Initially, an unadjusted difference and 95 % confidence interval will be calculated. A suitable GLM will then be fitted for each outcome. The logit link and binomial distribution will be used for binary outcomes, to identify link and Gaussian distribution for continuous outcomes, and log-link and Poisson distribution for count outcomes, which will include an overdispersion parameter if required,. Centre and baseline values will be included where appropriate as covariates in the models. The validated VFQ-25 will be used to score quality-of-life outcomes according to manual guidance, and the composite VFQ-25 score will be analysed as a continuous outcome. The CSRI and the EQ-5D-5L questionnaire will be analysed by the health economist.

#### Model assessment

For all methods outlined, goodness of fit will be checked using standard methods, such as the Hosmer-Lemeshow test to look at the discrepancy between observed and predicted values and residual plots to assess model assumptions. We will examine if there are any observations that have an undue influence on the model by looking at residual plots, and, where suitable, we will calculate Cook’s distance statistic. If model assumptions are not deemed to be valid or the model appears to be misspecified, we will undertake a sensitivity analysis with an alternative model.

### Adherence to allocated treatment and attrition

There is no monitoring of adherence, owing to the intervention and randomisation procedure, which occurs intraoperatively after the surgeon completes standard surgical procedures. For those randomised to the intervention arm, the investigational medicinal product is administered into the study eye immediately following randomisation.

Reasons for withdrawal from follow-up assessments will be summarised. Loss to follow-up over the 6 months is expected to be minimal, as there are only two data collection time points, both of which follow standard clinical care visits. It was assumed that no more than 7 % would be lost to follow-up, and this has been accounted for in the sample size of 300. The proportion of participants missing each of the primary and secondary outcomes will be summarised by each treatment arm.

### Harm data

Information on adverse events will be collected by means of spontaneous reports from patients and caregivers as well as clinical observation. No dictionary for coding adverse events will be used, and events will be coded using terms chosen by the clinical investigators. Adverse events, adverse reactions, serious adverse events and serious adverse reactions will be tabulated by allocated treatment arm for both the number of events and the number of participants with events. Along with the tabulation of the number of participants with raised IOP by treatment arm, the mean IOP and SD for each treatment group will be calculated for all participants by treatment arm. No formal statistical comparisons will be made.

## Conclusions

In this article, we describe the ASCOT trial statistical analysis plan and outline how data analysis will be performed. The protocol for the trial is published by Banerjee and colleagues [15]. We believe that, by making our statistical analytical plan visible and accessible, transparency regarding decisions made on the analysis of trial data will be maximised a priori, thus encouraging a balanced, accurate and complete reporting of results.

### Trial status

Recruitment started in December 2014 and is currently ongoing. We anticipate that the last patient will be followed in September 2017.

## Abbreviations

ASCOT, Adjunctive Steroid Combination in Ocular Trauma; CONSORT, Consolidated Standards of Reporting Trials; CSRI, Client Service Receipt Inventory; ETDRS, Early Treatment Diabetic Retinopathy Study, the standard scale to test visual acuity, which is based on letters of decreasing size on a chart; GLM, generalised linear model; IOP, intraocular pressure; KCTU, King’s Clinical Trials Unit; MAR, missing at random; OGT, open globe trauma; PVR, proliferative vitreoretinopathy; VA, visual acuity; VFQ-25, 25-item Visual Function Questionnaire

## Appendix 1: Baseline characteristics

The following baseline characteristics will be summarised by treatment arm:

1. Age
2. Sex
3. Ethnicity
4. Current smoker
5. Injury eye (left, right, both)
6. Ocular history condition (glaucoma, previous eye surgery, macular disease)
7. Cause of study eye injury
8. Previous primary repair
9. Classification of eye injury (rupture, penetrating, perforating, intraocular foreign body)
10. Extent of eye injury (zone 1, 2 or 3)
11. Presence of relative afferent pupil defect
12. Visual axis corneal scar
13. Uveitis
14. Hyphaema
15. Iris: normal, incomplete or incarcerated
16. Lens: clear, cataract, anterior or posterior chamber intraocular lens, aphakic intraocular lens
17. Vitreous haemorrhage
18. Endophthalmitis
19. ETDRS (total score or counting fingers/hand motion/light perception/no light perception)
20. Intraocular pressure
21. Retinal attachment (attached, traction retinal detachment, rhegmatogenous retinal detachment)
22. Fovea off (yes, no, splitting)
23. Presence of PVR
24. Presence of retinal incarceration
